# Energy Utilization of Building Insulation Waste Expanded Polystyrene: Pyrolysis Kinetic Estimation by a New Comprehensive Method

**DOI:** 10.3390/polym12081744

**Published:** 2020-08-05

**Authors:** Xiaoyang Ni, Zheng Wu, Wenlong Zhang, Kaihua Lu, Yanming Ding, Shaohua Mao

**Affiliations:** 1 Faculty of Engineering, China University of Geosciences, Wuhan 430074, China; yaotianyj@cug.edu.cn (X.N.); wu-zheng@cug.edu.cn (Z.W.); lukh@cug.edu.cn (K.L.); xyy@cug.edu.cn (S.M.); 2 State Key Laboratory of Coal Resources and Safe Mining, China University of Mining and Technology, Xuzhou 221116, China; 3 China Ship Development and Design Center, Wuhan 430064, China

**Keywords:** expandable polystyrene, pyrolysis, particle swarm optimization, kinetic parameters

## Abstract

Expanded polystyrene (EPS) has excellent thermal insulation properties and is widely applied in building energy conservation. However, these thermal insulation materials have caused numerous fires because of flammability. Pyrolysis is necessary to support combustion, and more attention should be paid to the pyrolysis characteristics of EPS. Moreover, pyrolysis is considered to be an effective method for recycling solid waste. Pyrolysis kinetics of EPS were analyzed by thermogravimetric experiments, both in nitrogen and air atmospheres. A new method was proposed to couple the Flynn–Wall–Ozawa model-free method and the model-fitting method called the Coats–Redfern as well as the particle swarm optimization (PSO) global algorithm to establish reaction mechanisms and their corresponding kinetic parameters. It was found that the pyrolysis temperature of EPS was concentrated at 525–800 K. The activation energy of EPS in nitrogen was about 163 kJ/mol, which was higher than that in air (109.63 kJ/mol). Furthermore, coupled with Coats–Redfern method, reaction functions *g(α) = 1 − (1 − α)^3^* and *g(α) = 1 − (1 − α)^1/4^* should be responsible for nitrogen and air reactions, respectively. The PSO algorithm was applied to compute detailed pyrolysis kinetic parameters. Kinetic parameters could be used in further large-scale fire simulation and provide guidance for reactor design.

## 1. Introduction

Among the many energy consumptions, the proportion of energy consumption in buildings is relatively large. Therefore, building energy conservation has attracted a lot of attention [1]. Researchers reached a consistent conclusion that buildings accounted for more than 30% of global energy consumption [2]. It can be seen that the needs of urban development will lead to continuous increasing energy consumption in buildings. Thus, it is a great necessity to improve energy efficiency and reduce energy consumption for buildings [3]. Therein, the important role of thermal insulation materials in improving building energy utilization is widely recognized [4]. In particular, expandable polystyrene (EPS) is widely employed in building exterior insulation systems [5], owing to its good thermal insulation performance, low cost, outstanding impact resistance, durability and so on [6].

With the increasing application scope of EPS and other polymers in different fields, a large amount of EPS is produced, and its waste volume is also huge from reconstruction and refurbishment [7]. Furthermore, EPS is not biodegradable [8] and is one of the most intractable substances to be treated in municipal solid waste [9]. There are many methods to dispose of solid waste, such as landfill [10], incineration [11] and pyrolysis [12]. However, EPS is generally not suitable for landfill and is also prohibited from incineration [9]. Chen et al. [13] noted that pyrolysis was considered to be an effective method for recycling polymer waste. Furthermore, Li et al. [14] pointed out that pyrolysis has become a sustainable and environmental-friendly approach to achieve valuable fuel from polymer. Meanwhile, in recent decades, extensive usage of exterior wall insulation materials of buildings has caused a considerable number of high-rise fires [15]. Therefore, more attention has been paid to the fire resistance of thermal insulation materials in the rapidly developing construction industry [16]. In general, pyrolysis is necessary to support combustion, and pyrolysis products can be used as fuel [17]. As a consequence, it will be helpful to understand the combustion performance of EPS by studying its pyrolysis characteristics [18] and provide guidance for the utilization of EPS waste in renewable energy [19]. Jiao et al. [20] carried out the EPS pyrolysis in nitrogen, and the results showed that EPS had only one stage of mass loss. Azimi et al. [21] calculated the kinetic parameters of EPS by iso-conversional methods. Kannan et al. [22] analyzed pyrolysis characteristics of EPS in various gaseous environments to reveal the different reaction mechanisms. However, these previous papers were still not enough to obtain detailed reaction mechanisms of EPS in different environments. Although Jun et al. [23] obtained kinetic parameters (activation energy *E_a_*, pre-exponential factor *A*, reaction order *n* and *m*) of EPS in various concentrations of oxygen, they emphasized that the objective of the study was to measure kinetic parameters rather than describe the reaction mechanisms. Therefore, it is necessary to establish the reaction mechanisms of pyrolysis.

All the mentioned studies about EPS pyrolysis were based on conventional iso-conversional methods. However, with the effective application of optimization algorithms in recent years, more and more researchers found that traditional iso-conversional methods were increasingly difficult to meet the needs of calculating pyrolysis kinetic parameters of high polymer materials [24]. Because kinetic parameters can better reflect the micro-scale pyrolysis process, they will improve understanding of thermochemical transformation technology, and provide valuable data and guidance for the design of pyrolysis and gasification equipment [25]. Moreover, Chauhan [9] noted that kinetic parameters were helpful to optimize the degradation process to obtain products with a maximum yield, which contributed to waste management. Meanwhile, they can be used for further large-scale fire simulation, such as cone calorimeter experiments [26] and the Fire Propagation Apparatus experiments [27]. Therefore, researchers tried to obtain pyrolysis kinetic parameters and explore the pyrolysis process based on the experimental data by using new efficient optimization algorithms, such as shuffled complex evolution (SCE) [28], genetic algorithm (GA) [13], particle swarm optimization (PSO) [29] and so on. Based on the advantages of PSO compared to GA by Ding et al. [30], PSO is applied in our current study.

The objective of this paper is to establish the detailed pyrolysis reaction mechanism of EPS in different environments. There are few studies to explore the pyrolysis of EPS under both nitrogen and air environments using a comprehensive approach (the model-free and model-fitting methods coupled with optimization algorithms). To further enhance the understanding of the thermal degradation of EPS in nitrogen and air, the Flynn–Wall–Ozawa model-free method and the Coats–Redfern model-fitting method are expected to predict reaction mechanisms and calculate kinetic parameters. Finally, based on the established mechanisms, PSO is employed to optimize kinetic parameters obtained by using the thermogravimetric data.

## 2. Materials and Methods

### 2.1. Materials

**Figure 1:**
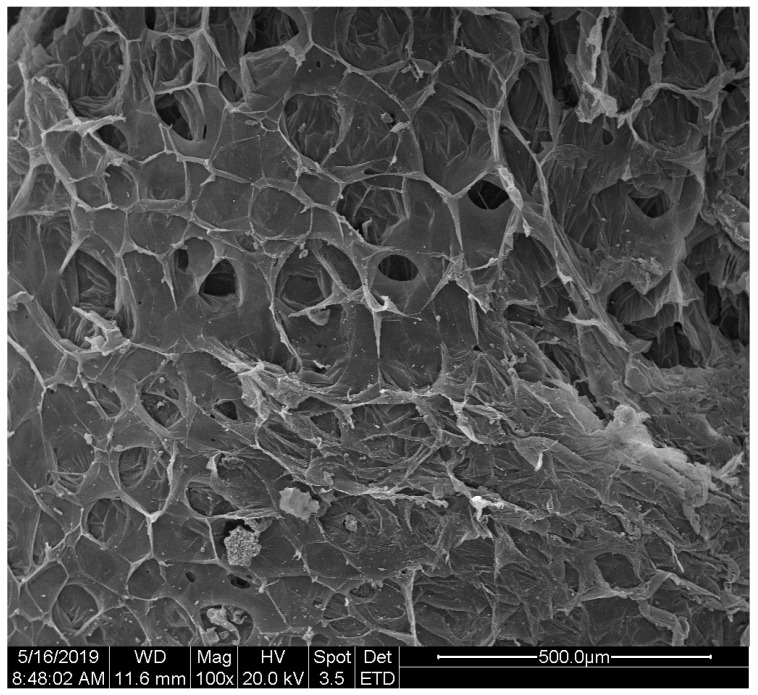
Scanning electron micrography of expanded polystyrene (EPS) enlarged 100 times.

EPS adopted in the present study is an amorphous, linear polymer, and its molecular weight is as much as 3.0 × 10^5^ to 5.0 × 10^5^ [31]. The elemental composition is measured by the Elementar Vario EL cube(Elementar, Langenselbold, Germany), which shows that EPS is composed of 89.69% carbon, 8.11% hydrogen and 2.2% nitrogen. EPS has many outstanding basic characteristics. Its thermal conductivity is 0.04 W/(mK) [32], which is good for thermal insulation. The density is 18 kg/m^3^ [33]. However, the value of limiting oxygen index (LOI) for EPS is 17.3% [34]. The typical microstructure of EPS enlarged 100 times is illustrated in Figure 1. It shows that the structure of EPS is spongy and cellular [35]. This structure can effectively use air to support combustion and make the flame spread quickly.

### 2.2. Thermogravimetric Measurements

Thermogravimetric analysis is a convenient and indispensable method to investigate pyrolysis kinetics [36]. The samples were milled into powder and dried for 24 h with a temperature of 80 °C before the experiments. The thermogravimetric experiment was carried out at a flow rate of 100 mL/min in nitrogen and air using a thermal analyzer (TA Instrument SDT Q600, New Castle, DE, USA), with a temperature range of 300–1000 K. The powdery specimen with a mass of approximately 5 mg was evenly distributed in an Alumina cup without a lid. Three heating rates including 5, 20 and 80 K/min were selected for pyrolysis.

### 2.3. Pyrolysis Kinetics

The reaction rate function of the pyrolysis process is defined by Equtions (1) and (2).

(1) $$\frac{d\alpha }{dt}=k\left(T\right)f\left(\alpha \right)$$

(2) $$\alpha =\frac{m_{0}-m_{t}}{m_{0}-m_{\infty }}$$

where *m_0_*, *m_t_* and *m_∞_* denote the specimen initial mass, actual mass and the final mass, respectively. *t* is time, *α* denotes the conversion rate and *f(α)* represents the differential function of the reaction model. *k(T)* can be shown as:

(3) $$k\left(T\right)=A\exp\left(\frac{-E_{a}}{RT}\right)$$

where *T* means the reaction temperature (K). *E_a_*, *A* and *R* represent the activation energy, pre-exponential factor and universal gas constant, respectively. Inserting heating rate *β*, Equation (4) can be represented as:

(4) $$\frac{d\alpha }{dT}=\frac{A}{\beta }f\left(\alpha \right)\exp\left(\frac{-E_{a}}{RT}\right)$$

### 2.4. Methods

#### 2.4.1. Flynn–Wall–Ozawa Method (FWO)

FWO [37,38] is a model-free method in integral form, and it is reliable to calculate the *E_a_* before knowing the reaction mechanisms [39]. The equation of the FWO method based on Doyle’s approximation [40] can be expressed as:

(5) $$\ln\beta =\ln\left(\frac{AE_{a}}{Rg\left(\alpha \right)}\right)-5.331-1.052\left(\frac{E_{a}}{RT}\right)$$

where *g(α)* is an integral function. From this equation, *E_a_* is obtained by a slope of ln*β* against 1*/T* at a certain *α*.

#### 2.4.2. Coats–Redfern Method (CR)

Compared with the FWO method, the CR method [41] is a model-fitting method that calculates the *E_a_* with a certain reaction mechanism [42]. The equation of the CR method can be written as:

(6) $$\ln\frac{g\left(\alpha \right)}{T^{2}}=\ln\left(\frac{AR}{\beta E_{a}}\right)-\frac{E_{a}}{RT}$$

Once *g(α)* is known, there is a straight line in the plot of ln(*g(α)/T^2^*) against 1*/T*, which makes it possible for the slope and intercept of the straight line to determine *E_a_* and *A*. The reaction mechanisms during the solid-state process are proposed in Table 1.

#### 2.4.3. Global Optimization Method-PSO

The PSO algorithm, as a heuristic algorithm, is commonly used in pyrolysis with some advantages, such as simplifying the difficulty of calculation, saving time and improving accuracy and efficiency of calculation.

The PSO algorithm has two search models (velocity and position) of numerous particles. The objectives of velocity and position are employed to update the position of the particle and represent a candidate solution in space [29,45]. Assuming that the number of particles is *n* in the *D*-dimensional search space, the velocity vector of the *i*th particle is expressed as *v_i_* = (*v_i1_*,*v_i2_*,...,*v_iN_*), and the position vector *x_i_* = *(x_i1_,x_i2_,...,x_iN_)*. The fitness value of the particles is calculated by the following functions:

(7) $$\phi =\phi_{m}+\phi_{mlr}+\phi_{\alpha }+\phi_{d\alpha /dt}$$

(8) $$\phi_{m}=\sum_{j=1}^{N}\left[w_{CML,j}\frac{\sum_{k=1}^{n}{\left(CML_{\mathrm{mod},k}-CML_{\exp,k}\right)}^{2}}{\sum_{k=1}^{n}{\left(CML_{\exp,k}-\frac{1}{n}\sum_{p=1}^{n}CML_{\exp,p}\right)}^{2}}\right]$$

(9) $$\phi_{mlr}=\sum_{j=1}^{N}\left[w_{MLR,j}\frac{\sum_{k=1}^{n}{\left(MLR_{\mathrm{mod},k}-MLR_{\exp,k}\right)}^{2}}{\sum_{k=1}^{n}{\left(MLR_{\exp,k}-\frac{1}{n}\sum_{p=1}^{n}MLR_{\exp,p}\right)}^{2}}\right]$$

(10) $$\phi_{\alpha }=\sum_{j=1}^{N}\left[w_{\alpha ,j}\frac{\sum_{k=1}^{n}{\left(\alpha_{\mathrm{mod},k}-\alpha_{\exp,k}\right)}^{2}}{\sum_{k=1}^{n}{\left(\alpha_{\exp,k}-\frac{1}{n}\sum_{p=1}^{n}\alpha_{\exp,p}\right)}^{2}}\right]$$

(11) $$\phi_{d\alpha /dt}=\sum_{j=1}^{N}\left[w_{d\alpha /dt,j}\frac{\sum_{k=1}^{n}{\left({d\alpha /dt}_{\mathrm{mod},k}-{d\alpha /dt}_{\exp,k}\right)}^{2}}{\sum_{k=1}^{n}{\left({d\alpha /dt}_{\exp,k}-\frac{1}{n}\sum_{p=1}^{n}{d\alpha /dt}_{\exp,p}\right)}^{2}}\right]$$

where *Φ_m_*, *Φ_mlr_*, *Φ_α_* and *Φ_dα/dt_* refer to the objective function for mass loss, mass loss rate, conversion rate and reaction rate, respectively. *N* denotes the number of experiments. *CML* is the cumulative mass loss, *MLR* is the cumulative mass loss rate and *α* and *dα/dt* represent the cumulative conversion rate and reaction rate, respectively. *λ* represents the number of experimental data points with each experiment, and *w* is the weighted value. Subscript *mod* and *exp* are the modeled and experimental values.

In addition, particles have the memory ability to keep the optimal position of personal (*p_i_*) and global position (*p_g_*). Before searching, the particle has an assigned value with a rational range. Next are the updated formulas of the particles via the following equations:

(12) $$v_{id}^{k+1}=wv_{id}^{k}+c_{1}r_{1}\left(p_{id}-x_{id}^{k}\right)+c_{2}r_{2}\left(p_{gd}-x_{id}^{k}\right)$$

(13) $$x_{id}^{k+1}=x_{id}^{k}+v_{id}^{k+1}$$

where *k* stands for the iteration number, *i* denotes the number of the particles and *d* represents the search direction. *w* is a constant called inertia weight. *c*_1_ and *c*_2_ are defined constants, and *r*_1_ and *r*_2_ are constants in the range [0, 1].

## 3. Results and Discussion

### 3.1. Thermogravimetric Analysis

**Figure 2:**
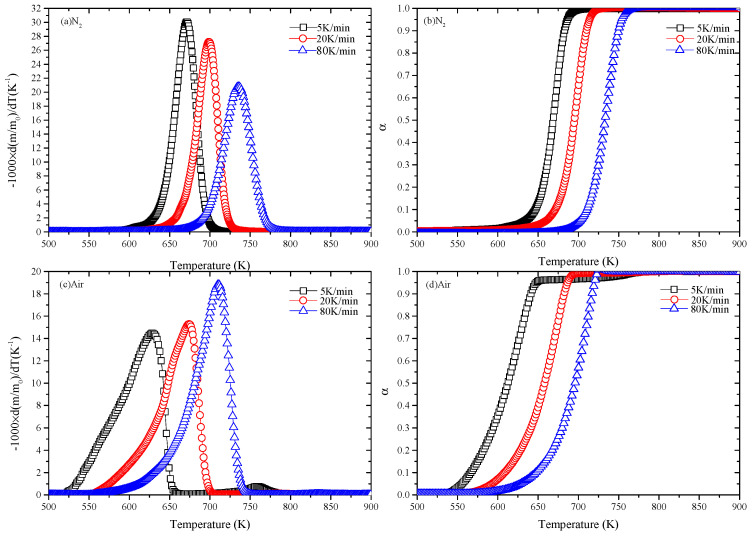
DTG and the conversion rate curves in nitrogen and air: (**a**) DTG (nitrogen), (**b**) *α* (nitrogen), (**c**) DTG (air) and (**d**) *α* (air).

Figure 2 precisely illustrates the changes in the derivative mass loss (DTG) and the conversion rate curves with the temperature at three heating rates (5, 20, 80 K/min) in nitrogen and air. Figure 2a,c shows the DTG curves of EPS samples in nitrogen. The pyrolysis reaction of EPS mainly occurs between the temperatures of 525 and 775 K. It is evident that the variation tendencies of peak locations are also consistent: the slower the heating rate is, the lower the temperature of peak location is. Moreover, only one peak exists for each DTG curve. However, the pyrolysis behavior of EPS in the two environments is different. The temperatures corresponding to peak locations in nitrogen are higher than that in air. Liu et al. [46] proposed an empirical formula for the *T_p_* versus *β*, which can be expressed as:

(14) $$T_{p}=365.1\beta^{0.0565}$$

where *T_p_* is the peak temperature. The value of *T_p_* is calculated by Eqution (14) and compared with the experimental temperature. Table 2 illustrates that the calculated peak temperature is much in agreement with the experimental temperature in nitrogen.

Furthermore, the DTG curves for EPS in nitrogen show that the peak values in nitrogen are higher than that in air. Therein, peak values are 30, 27 and 21 K^−1^ in nitrogen, while in air are 14.5, 15 and 19 K^−1^, which means that the higher heating rates are, the closer the peak values of DTG curves are. Furthermore, EPS starts to degrade at 600 K in nitrogen, which is much higher than in air (525 K). The reaction temperature range of the three heating rates in nitrogen is about 100 K and 150 K in air, and the curve in nitrogen is steeper than that in air, which indicates that the reaction time is shorter in nitrogen. The shift of conversion rate curves is shown in Figure 2b,d. It illustrates that the higher the heating rate, the higher the reaction temperature.

### 3.2. Kinetic Analysis by the FWO Method

The *E_a_* value is obtained according to the formula (−1.052*E_a_/R*) versus 1/*T*, and it is shown in Table 3. Table 3 shows that the differences in *E_a_* values of EPS in nitrogen are very small, and the average value remains almost constant at about 163.23 kJ/mol. For EPS in air, the value of *E_a_* increases slowly from 98 to 125 kJ/mol during pyrolysis, but the difference between the maximum and minimum values is less than 30% of the average value, and the average value of *E_a_* is 109.85 kJ/mol, which is lower than that in nitrogen.

The reason for the different *E_a_* of EPS in nitrogen and air is that the reaction mechanism is different in the two environments. Furthermore, the thermal degradation of EPS is more complex in air. If oxygen is present in the pyrolysis process, it will react with solid reactants, which is a heterogeneous reaction. Meanwhile, homogenous reactions will be generated between oxygen and volatiles [47]. Kannan et al. [22] indicated that the mechanism of EPS degradation in air was that polymer radicals were generated first, and oxygen of air reacted with polymer radicals, so a peroxy radical intermediate was formatted. Then, more polymer radicals were produced due to the decomposition of peroxy radicals and acceleration of the oxidation process, which lasted until the end of the reaction.

### 3.3. Establish Reaction Mechanisms

The *E_a_* values are calculated based on thermogravimetric data by the CR method and presented in Table 4. If the established pyrolysis mechanism can reflect the pyrolysis process of EPS, the *E_a_* calculated by FWO should be the closest to that obtained by the CR method [48]. For EPS in nitrogen, the estimated average *E_a_* for the cases of the three heating rates varies from 51.28 to 532.55 kJ/mol for all the mentioned in our current study. Therein, the *E_a_* value in nitrogen corresponding to the reaction function *g(α) = 1 − (1 − α)^3^* is about 158.17 kJ/mol (*R^2^* = 0.902), which is close to the calculated value (163.23 kJ/mol) of the FWO method. While for EPS in air, the estimated *E_a_* is between 44.99 and 219.33 kJ/mol. Furthermore, the *E_a_* value of *g(α) = 1 − (1 − α)^1/4^* is 105.14 kJ/mol (*R^2^* = 0.996), which is basically the same with 109.85 kJ/mol. In addition, the corresponding logarithmic form (ln*A*) of the pre-exponential factors can be obtained by a certain reaction mechanism and, which is listed in Table 3.

The value of ln*A* is similar to the changing trend of *E_a_*, that is, ln*A* remains stable in nitrogen, while it gradually increases in air. The reason for this phenomenon is that there is a linear relationship between *E_a_* and ln*A*, which is applied to verify whether the established reaction mechanism is appropriate [49]. The linear relationship can be written as:

(15) $$\ln A=aE_{a}+b$$

where *a* and *b* refer to constants.

The relationships in nitrogen and air of Eqution (15) are ln*A* = 0.17*E_a_* − 4.72 (*R^2^* = 0.919) and ln*A* = 0.20*E_a_* − 7.34 (*R^2^* = 0.999), respectively. Based upon the above discussion, the reaction functions of EPS in nitrogen are *g(α) = 1 − (1 − α)^3^* and *g(α) = 1 − (1 − α)^1/4^* in air. Therefore, Eqution (4) can be expressed as:

(16) $$\frac{d\alpha }{dt}=A{\left(1-\alpha \right)}^{n}\exp\left(\frac{-E_{a}}{RT}\right)$$

In order to obtain the detailed kinetic parameters pre-exponential factor (ln*A*), activation energy (*E_a_*), reaction order (*n*) and char yield (*v*) for EPS pyrolysis, PSO is applied here.

### 3.4. Parameters Optimization by PSO

Thermogravimetric data at 5, 20 and 80 K/min are chosen to optimize by PSO. The initial values of ln*A*, *E_a_*, and *n* are 23.70 [ln(s^−1^)], 158.17 kJ/mol and −2 in nitrogen, and 14.11 [ln(s^−1^)], 105.14 kJ/mol and 3/4 in air, respectively. The kinetic model parameters (ln*A*, *E_a_*) are set at a wide search range of 50% to 150% of the initial values. For the reaction orders, the search range is set to −5 ≤ *n* ≤ 5. The value of *v* is 0.026 and 0.032 in nitrogen and air, and the search range is restricted to 0.05–0.95 [13]. The population size calculated by PSO is set to 2.5 × 10^3^, and the generation number is 1.0 × 10^4^. Table 5 shows the optimized values of the kinetic model parameters.

**Figure 3:**
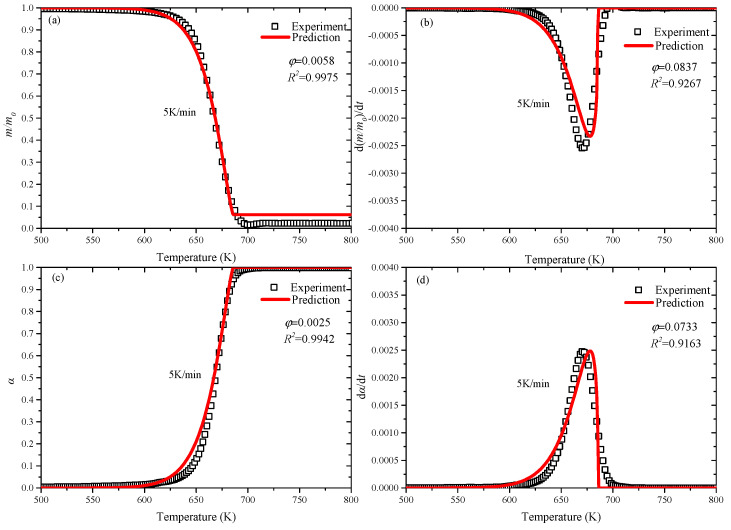
Predicted and experimental curves in nitrogen at 5 K/min: (**a**) *m/m_0_*; (**b**) *d(m/m_0_)/dt*; (**c**) *α*; (**d**) *dα/dt*.

**Figure 4:**
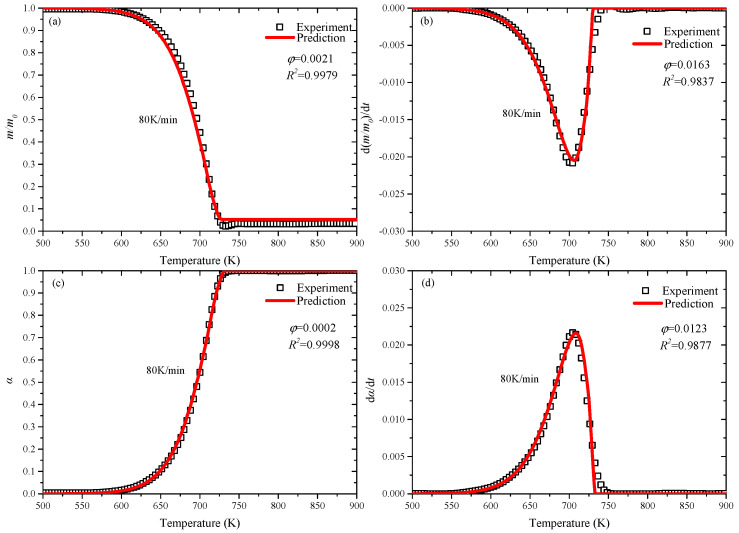
Predicted and experimental curves in air at 80 K/min: (**a**) *m/m_0_*; (**b**) *d(m/m_0_)/dt*; (**c**) *α*; (**d**) *dα/dt*.

In Figure 3 and Figure 4, the experimental data and predicted results in both environments represent a good agreement. With the application of PSO, numerical prediction of complex kinetics of reactants and products can be performed. Figure 3 and Figure 4 show the comparison of the predicted and experimental curves of mass loss (*m/m_0_*), derivative mass loss (*d(m/m_0_)/dt*), conversion rate (*α*) and reaction rate (*dα/dt*) in nitrogen and air.

Figure 3 shows that the predicted results at 5 K/min heating rate in nitrogen, the trend of predicted values and experimental data of *m/m_0_* and *α* are basically consistent. Furthermore, the *R*-squared values of *m/m_0_* and *α* curves are close to 1, and the values of *d(m/m_0_)/dt* and *dα/dt* exceed to 0.91. However, the deviation of *d(m/m_0_)/dt*, *dα/dt* are existing, and the peak values of prediction and experiment are fine agreement. Figure 4 shows the comparison at 80 K/min heating rate in air, and the agreement of all the predicted values is better than that in nitrogen, and the *R*-squared values are higher. PSO has achieved good optimization values at both lower (5 K/min) and higher (80 K/min) heating rates, so it can be applied to a wider range to predict kinetic model parameters of other heating rates.

## 4. Conclusions

Through thermogravimetric experiments at heating rates of 5, 20 and 80 K/min, the thermal degradation of EPS both in nitrogen and air atmospheres was investigated. Then coupling a model-free method called Flynn–Wall–Ozawa and the Coats–Redfern model-fitting method was used to obtain pyrolysis kinetic parameters. The two reaction functions of EPS pyrolysis were *g(α) = 1 − (1 − α)^3^* in nitrogen and *g(α) = 1 − (1 − α)^1/4^* in air to characterize the pyrolysis process. The established kinetic reaction model was coupled to a global optimization algorithm called particle swarm optimization to obtain model parameters that could be used in further large-scale fire simulation. It was found that predicted mass loss (*m/m_0_*) curves, derivative mass loss (*d(m/m_0_)/dt*) curves, conversion rate (*α*) curves and reaction rate (*dα/dt*) curves at various heating rates agreed well with experimental data.

## Figures and Tables

**Table 1:** Reaction mechanisms during the solid-state process [43,44].

*g(α)*	*f(α)*	Rate-Determining Mechanism
*α*	1	Contracting disk
1 − (1 − *α*)^1/2^	2(1 − *α*)^1/2^	Contracting area
1 − (1 − *α*)^1/3^	3(1 − *α*)^2/3^	Contracting volume
1 − (1 − *α*)^1/4^	4(1 − *α*)^3/4^	Chemical reaction
1 − (1 − *α*)^2^	1/2(1 − *α*)^−1^	Chemical reaction
1 − (1 − *α*)^3^	1/3(1 − *α*)^−2^	Chemical reaction
*α* ^2^	1/(2*α*)	1-*D* diffusion
(1 − *α*)ln(1 − *α*) + *α*	[−ln(1 − *α*)]^−1^	2-*D* diffusion
[1 − (1 − *α*)^1/3^]^1/2^	6(1 − *α*)^2/3^[1 − (1 − *α*)^1/3^]^1/2^	3-*D* diffusion

**Table 2:** Comparison of calculated peak values of EPS and experimental temperature using an empirical formula.

*β* (K/min)	Peak Temperature (K)			
	Calculated Values	Kannan et al. [22]	Experimental Values (N_2_)	Experimental Values (Air)
5	672	673	671	628
20	705	703	699	674
80	741	-	735	710

**Table 3:** The values of *E_a_* and ln*A* of the Flynn–Wall–Ozawa (FWO) method.

*α*	N_2_			Air		
	*E_a_* (kJ/mol)	*R^2^*	ln*A* [ln(s^−1^)]	*E_a_* (kJ/mol)	*R^2^*	ln*A* [ln(s^−1^)]
			*g(α) = 1 − (1 − α)^3^*			*g(α) = 1 − (1 − α)^1/4^*
0.10	153.79	0.979	21.91	98.83	0.993	11.84
0.20	162.32	0.985	23.63	98.97	0.986	12.00
0.30	162.32	0.985	23.68	102.50	0.982	12.72
0.40	163.64	0.987	23.91	106.97	0.982	13.60
0.50	164.98	0.988	24.10	110.94	0.985	14.37
0.60	166.33	0.989	24.27	115.08	0.986	15.15
0.70	166.10	0.986	24.11	120.24	0.984	16.13
0.80	166.33	0.989	24.02	125.20	0.986	17.06
Average	163.23	0.986	23.70	109.85	0.986	14.11

**Table 4:** The *E_a_* values of EPS both in nitrogen and air atmospheres by the Coats–Redfern (CR) method.

Reaction Model	Average Value (N_2_)		Average Value (Air)	
*g(α)*	*E_a_* (kJ/mol)	*R^2^*	*E_a_* (kJ/mol)	*R^2^*
*α*	238.00	0.985	95.03	0.995
1 − (1 − *α*)^1/2^	274.12	0.994	85.64	0.998
1 − (1 − *α*)^1/3^	287.34	0.995	86.99	0.997
1 − (1 − *α*)^1/4^	295.74	0.996	105.14	0.996
1 − (1 − *α*)^2^	182.05	0.952	66.61	0.969
1 − (1 − *α*)^3^	158.17	0.902	62.08	0.918
*α* ^2^	371.42	0.986	160.76	0.996
(1 − *α*)ln(1 − *α*) + *α*	532.55	0.992	219.33	0.998
[1 − (1 − *α*)^1/3^]^1/2^	138.70	0.995	52.60	0.996

**Table 5:** Optimized parameters and searing range by particle swarm optimization (PSO) on three heating rates.

Gas	Parameters	Search Range	Optimized Values	Integral Method [23]	Differential Method [23]
N_2_	ln*A* [ln(s^−1^)]	[10.96,36.41]	24.20	35.59	25.99
	*E_a_* (kJ/mol)	[76.90,250.89]	170.10	153.48	136.26
	*n*	[−5,5]	0.58	0.5	0.88
	*v*	[0.05,0.95]	0.05	-	-
Air	ln*A* [ln(s^−1^)]	[5.92,25.59]	16.58	22.47	22.62
	*E_a_* (kJ/mol)	[49.19,187.80]	121.47	104.31	126.52
	*n*	[−5,5]	0.45	0.5	0.77
	*v*	[0.05,0.95]	0.16	-	-
